# Performance Analysis of a Defected Ground-Structured Antenna Loaded with Stub-Slot for 5G Communication

**DOI:** 10.3390/s19112634

**Published:** 2019-06-10

**Authors:** Md Mushfiqur Rahman, Md Shabiul Islam, Hin Yong Wong, Touhidul Alam, Mohammad Tariqul Islam

**Affiliations:** 1 Faculty of Engineering, Multimedia University, Persiaran Multimedia, Cyberjaya 63100, Selangor, Malaysia; shabiul.islam@mmu.edu.my (M.S.I.); hywong@mmu.edu.my (H.Y.W.); 2 Centre of Advanced Electronic and Communication Engineering (PAKET), Faculty of Engineering and Built Environment, University Kebangsaan Malaysia (UKM), Bangi 43600, Selangor, Malaysia; touhid13@siswa.ukm.edu.my

**Keywords:** 5G, defected ground structure, electronic band gap, stub-notch configuration

## Abstract

In this paper, a defected ground-structured antenna with a stub-slot configuration is proposed for future 5G wireless applications. A simple stub-slot configuration is used in the patch antenna to get the dual band frequency response in the 5G mid-band and the upper unlicensed frequency region. Further, a 2-D double period Electronic band gap (EBG) structure has been implemented as a defect in the metallic ground plane to get a wider impedance bandwidth. The size of the slots and their positions are optimized to get a considerably high impedance bandwidth of 12.49% and 4.49% at a passband frequency of 3.532 GHz and 6.835 GHz, respectively. The simulated and measured realized gain and reflection coefficients are in good agreement for both operating bandwidths. The overall antenna structure size is 33.5 mm × 33.5 mm. The antenna is fabricated and compared with experimental results. The proposed antenna shows a stable radiation pattern and high realized gain with wide impedance bandwidth using the EBG structure, which are necessary for the requirements of IoT applications offered by 5G technology.

## 1. Introduction

The world is going to experience 5G technology very soon with the highest broadband speed and minimum latency. To ensure widespread coverage and support for all services covered by the previous technology, it needs to work with three frequency ranges. These are Sub-1 GHz, 1–6 GHz, and above 6 GHz. The Sub-1 GHz band is used for providing widespread coverage in the cities, suburban, and village areas and help to support the Internet of thing services. On the other hand, the 1–6 GHz band provide both capacity and coverage advantages. The band above 6 GHz fulfills the requirements of an ultra-high broadband speed of 5G. According to the report by Global mobile Suppliers Association (GSA), a substantial number of countries are considering, planning, or in the process of auctioning the band around 3.5 GHz. However, to meet the requirement of high broadband speed of up to 20 Gb/s, there will be a need to utilize millimeter wavebands starting from 24 GHz. GSMA recommended 26 GHz (24.25 GHz–27.5 GHz), 40 GHz (37.5 GHz–43.5 GHz), and 67 GHz–71 GHz bands for the mobile. For this reason, auctions will take place in the following years in many countries to license the 5G mid-bands and millimeter wavebands. The operators could also utilize the unlicensed bands to extend the 5G user experience by aggregating it with the licensed one. So for the 5G mid-band of 3.5 GHz, the unlicensed band can be of 6 GHz frequency which is similar to the Wi-Fi bands of 2.4 GHz and 5 GHz; the 2.4 GHz band is used for the coverage, and 5 GHz is used for the high data rate. Similarly, the 3.5 GHz band could be used to cover coverage and capacity, i.e., it will handle the increasing number of subscribers, and the unlicensed 6 GHz band could be used to provide high data rate. So, designing patch antennae resonating in these frequency bands will be a prudential step [1,2].

In addition, designing antennae for 5G communications should consider some key requirements of 5G technology. First of all, to experience the fastest 5G services, regulators should assign at least 100 MHz per operator in the 5G mid-band and 1 GHz in millimeter wavebands. So, the designed antenna should resonate over a wider bandwidth covering the whole license band but avoid the frequency bands that are already in use for 4G. Secondly, to get the benefits of services like autonomous car driving, augmented reality, virtual reality, and tactile internet which are highly demanding services of 5G communication, continuous strong connections with minimum latency are required. To fulfill this requirement, the antenna should possess high gain. Last of all, because the beginning of the 5G technology will experience a massive number of small IoT, a compact antennae design for these tiny devices will be essential [3]. Defected Ground Structure (DGS) is one of the popular techniques that is used to enhance the patch antenna performance by increasing the gain and bandwidth, lowering cross-polarization, and coupling in the case of the array antenna.

The DGS structure is similar to a unit cell or a periodic configuration which is placed directly under the transmission line [4,5]. But recent research has shown that the placement of this structure at a sliding displacement from the underneath position of the feed line can also lead to a good parametric output. Some methods are used to reduce cross-polarization [6,7,8], and some others have focused on the improvement of other parameters like gain, bandwidth, and return loss [9,10,11,12,13]. Good isolation between the co- and cross-polarization (XP), as well as reduced XP levels in the H-plane, have been achieved by implementing symmetric and non-proximal DGS shapes in [6], but a slight gain reduction was observed. Whereas the proximal position of a composite type of DGS structure leading to a reduction of XP and also a reduction in the back radiation was achieved in [7]. On the other hand, two circular slots were used to reduce XP in [8]. In [9], a circular dumbbell-Shaped DGS was implemented to increase the bandwidth of all three bands, but at the same time, the gain was slightly decreased. In [10], two different but complimentary DGS structures were implemented which lead to increased bandwidth as well as reduced return loss. In [11,12], rectangular and H slots respectively were used to achieve improved parameters. Both produced enhanced bandwidth and gain in all the three bands, but the efficiency dropped. In [13], good patch miniaturization was achieved using a shorting post, and a U shaped DGS; however, the lower band had poor gain and efficiency. In [14], a tri-band antenna was presented where miniaturization was achieved by implementing an E-type unit cell in conjunction with an F shaped slot as a DGS but resulted in a narrow bandwidth. Other design approaches where multiband operations are achieved but with narrow bandwidth characteristics also exist. In [15], a dual-band antenna was designed where miniaturization was achieved by etching a square slot in the middle of the patch, and a pair of mirror image L-shaped slots with a pair of slits were cut on the radiating element to achieve the dual-band operation but produced a narrow bandwidth. In [16], a U-shaped feeding method was used to increase the gain but the bandwidth was not so promising. In [17], a rounded corner rectangle radiating patch was used to achieve a dual-band characteristic and high gain but with a narrow bandwidth. DGS can also be used to achieve band notch characteristics. A pair of DGS resonators have been used to reject the downlink of X-band satellite communications (7.0–7.40 GHz) [18]. This rejection can be controlled by the gap width of the resonator. On the other hand, by introducing an open ring DGS [19] on the metasurface unit, a high lower-stopband suppression level of 20 dB is achieved while the in-band antenna performance remains unaffected. However, the antenna size is bigger for the 5G IoT applications. DGS has also proved its importance in reducing mutual coupling between array antennae. An octagonal ring slot [20] with cross lines has been implemented as a DGS unit to enhance the isolation of a 2 × 2 multiple-input and multiple-output (MIMO) antenna, but the impedance bandwidth is a narrow bandwidth.

This paper provides an analysis of the effect of loading defected metallic ground planes with a simple stub-slot configuration. The defects are etched symmetrically but not underneath the feed line and are also totally isolated from each other, i.e., they are not connected by a split line to form a conventional dumbbell-shaped DGS, so, the structure is similar to a 2-D multiperiod electronic structure. However, the radiating element of the patch is modified by the combination of stubs and slots. This combination is responsible for the dual band nature in the desired frequency spectrum. Further, the addition of DGS results in a wider bandwidth with little effect on the position of the passband frequencies.

## 2. Design and Analysis

**Figure 1:**
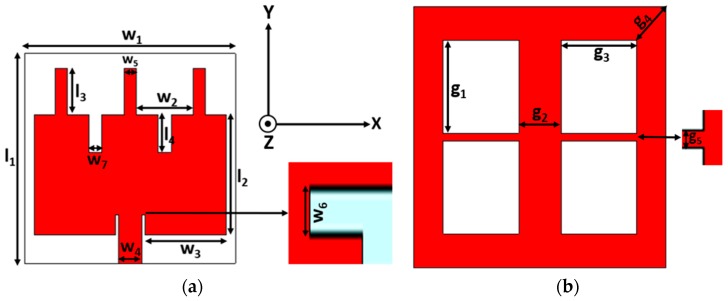
Schematic of the microstrip line edge-fed rectangular patch. (**a**) Top view with the stub loaded and slot etched rectangular patch; (**b**) Back view of the defected ground structure (DGS)-integrated ground plane.

The proposed antenna is designed on a Rogers RT/Duriod 6202 with relative permittivity 2.94 and loss tangent of 0.0015. All the patch elements are laminated on the top of the substrate, and the ground plane is on the opposite side of the patch where four rectangular slots are placed as DGS elements. The overall dimension is 33.5 mm × 33.5 mm × 1.52 mm which is depicted in Figure 1. The corresponding values are listed in Table 1.

### 2.1. Stub-Notch Configuration Analysis

Open-circuit stubs are loaded with the radiating edge of the antenna. A dual-band frequency response is achieved with this configuration. Without loading the stubs, the inset-fed antenna gives only one −10 dB resonance and two band notches in the higher bands. According to [21,22,23], placing an open circuit stub with a length nearly equal to a quarter of the wavelength on the radiating edge, can offer a capacitive impedance for the frequencies lower than the patch resonant frequency, and inductive impedance for the frequencies higher than the patch resonant frequency which leads to a dual frequency response. But for different stub lengths, this quarter wavelength approximation does not give exact results for the given resonant frequency [23]. Moreover, the position of the stub on the radiating edge changes the impedance variation along the edge. So, to have appropriate impedance matching, the feed position should be near the area of the opposite edge of the stub, and for further improvement, the feed position can be displaced from the y-axis position where the stub is present [24]. In the present work, a stub of length not equal to quarter the wavelength has been loaded on the radiating edge which gives two −10 dB resonance but does not affect the middle-band notch. Though its length is not equal to a quarter of the wavelength, it still offers capacitive impedance to the lower resonance frequency and inductive impedance to the higher resonance frequency. For this reason, both −10 dB resonance positions shift to the lower frequency regions, but the lower passband frequency (3.96 GHz) is not the desired frequency for this application.

**Figure 2:**
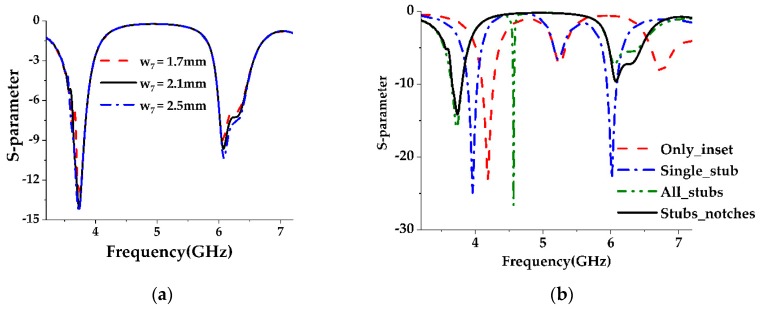
Parametric analysis of the stub-notch configuration. (**a**) Effect of the notch width (w_7_) on the passband frequencies; (**b**) Effect of the stub-notch configuration on the passband frequencies.

So, we need to further shift the passband frequencies to lower frequency regions. Loading of additional stubs whose lengths are not equal to a quarter of the wavelength and symmetric to the middle stub has the same effect of loading a single stub, i.e., offering more capacitive and inductive impedance to the lower and higher resonant frequencies, respectively. But loading of these stubs forms a coupling capacitance between each of them and the center stub. So, they are responsible for another resonant frequency, and a spike is observed between the two resonant frequencies achieved earlier. In addition, the impedance matching of the previous passband frequencies degrade. To solve this problem, instead of changing the position of the feed line, a pair of rectangular slots are cut in between these three stubs and from the same radiating edge. These rectangular slots can be referred to as notches. The loading of these notches increases the current path at the radiating edges where the stubs are located, which further increases the inductive impedance offered by the stubs. At the same time, the notches will slightly increase the value of the coupling capacitance. For the negligible value of coupling capacitance with respect to the inductive impedance, the resonance behavior of the side stubs diminishes. On the other hand, the widths of these notches have little effect on the position of the resonance frequencies [24] as depicted in Figure 2a.

**Figure 3:**
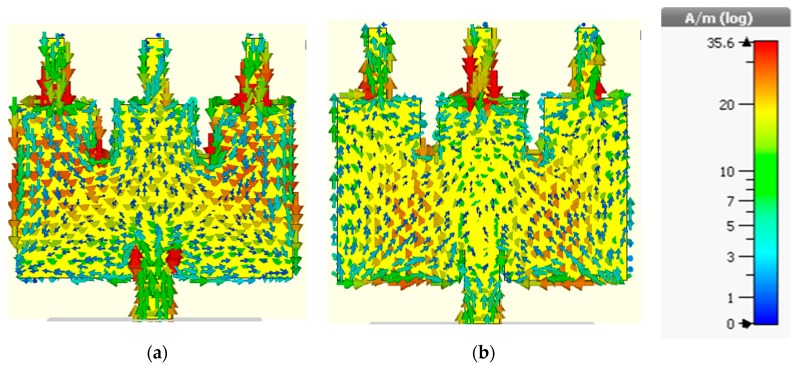
The current distribution of the antenna without DGS. (**a**) 3.736 GHz; (**b**) 6.0734 GHz.

The distribution of the stub-notch configuration is presented in Figure 3. At a lower passband frequency (3.736 GHz), the current bends at the inset’s upper side corners and gathers along the edges of the patch and then follows a straight path to the side stubs. So, little current flows around the center stub rather than the side stubs. As current flows in a more curved path at higher frequencies, the current changes its direction at the higher passband frequency (6.07 GHz), after passing the inset picks and the edges followed by a curved path towards the center stub.

### 2.2. DGS Structure Analysis

The concept of the defected ground structure (DGS) originated from the research on the photonic bandgap structure (PBG). The name of the concept “DGS” simply means that a defect has been in the ground plane which could be considered as an approximation of an infinite and perfectly conducting current sink. It is of a compact geometry which is commonly known as a unit cell that is etched out in the backside metallic ground plane as a single defect or a periodic configuration. This artificial structure prevents the electromagnetic (EM) waves from propagating through the defects over a range of frequencies (stopband), but allow the EM waves to pass through them (defects) over a range of frequencies (passband). That’s why PBG’s implemented in EM applications are also referred to as electromagnetic band gap (EBG) structures. However, this nature of influencing the guided wave characteristics produces bandgap properties and slow wave effects which help provide more compact printed circuits.

In terms of current distribution, the presence of these slots under the printed transmission line disturbs the current distribution in the ground plane. This leads to a reduction in the image current, and therefore, the surface wave reduces. Moreover, due to the reduction of the surface wave, the stored energy as well as the Q factor decreases and results in bandwidth enhancement.

Previously, a number of DGS structures including unit cells and periodic configurations with 1-D, 2-D, or with uniform or non-uniform structures have been studied for various purposes such as the implementation of filters, suppression of unwanted surface waves, and control of harmonics in microstrip antennas, to achieve compactness and improve performance in terms of stopbands and passbands. In this paper, a 2-D multiperiod EBG is analyzed with the stub-notch configuration to study the effect of this structure on the antenna performance.

In the proposed design, the effect of loading a 2-D EBG with two periods on the stub-notch configuration has been studied and compared with the dumbbell shaped DGS. The dimensions of the EBG structure are generated by using the conventional equations in [24].

**Figure 4:**
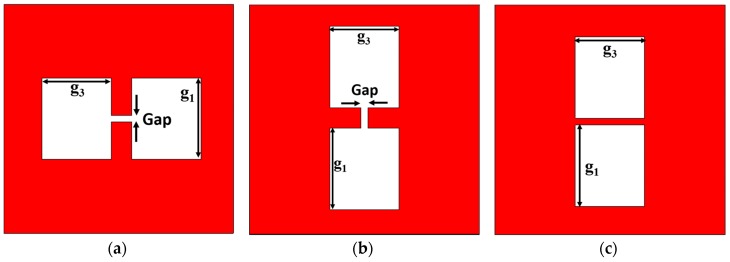
Different DGS configurations. (**a**) Parallel dumbbell-shaped unit cell; (**b**) Perpendicular dumbbell-shaped unit cell; (**c**) Proposed electromagnetic band gap (EBG) structure.

**Figure 5:**
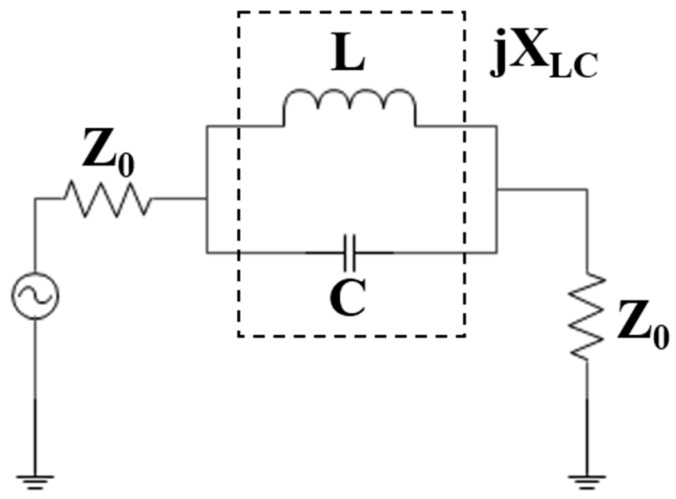
An equivalent circuit to the Dumbbell-shaped DGS. Z_0_ denotes the scaled impedance level of the in/out terminated ports.

The equivalent circuit of a pair of rectangular EBG lattice shapes etched in the metallic ground plane parallel with respect to the transmission line can be obtained from the equivalent circuit of a dumbbell-shaped lattice as shown in Figure 4a [25]. The equivalent circuit of a dumbbell-shaped lattice etched perpendicularly in the ground plane with respect to the transmission line is shown in Figure 5 which is a parallel LC resonator. For this type of unit cell, the increased lattice area increases the series inductance of the transmission line and so the resonance shifts to a lower frequency. The variation of the gap distance has little effect on the passband frequency but affects the attenuation pole location. The change in attenuation pole location means that there is a parallel capacitance (C) with a series inductance (L) that is provided by the gap distance [26].

**Figure 6:**
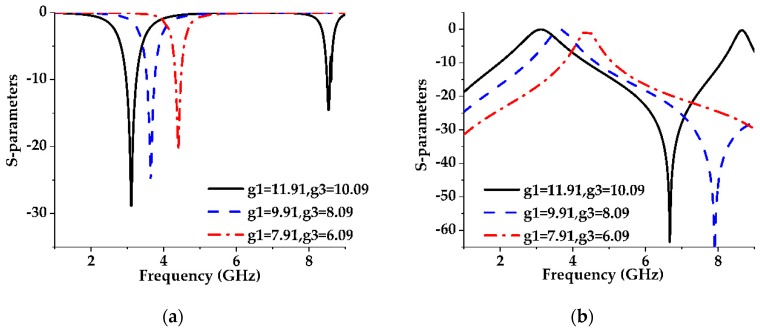
Effect of the lattice size on the S-parameters of the parallel dumbbell-shaped DGS unit cell with a constant gap width of 1 mm. (**a**) Reflection coefficient (S11) (**b**) Transmission coefficient (S21).

**Figure 7:**
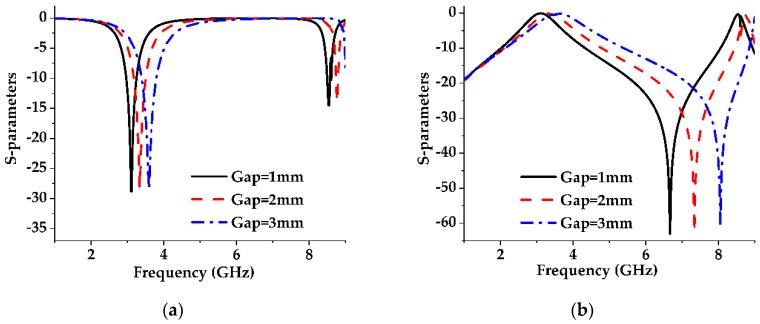
The effect of the gap width on the S-parameters of the parallel dumbbell-shaped DGS unit cell with a constant lattice size of g1 = 11.91 mm and g3 = 10.09 mm. (**a**) S11; (**b**) S21.

The same analysis has been performed on the dumbbell-shaped lattice which is placed parallel with respect to the transmission line as shown in Figure 4b and got the same results, shown in Figure 6 and Figure 7 as for the dumbbell-shaped DGS unit perpendicularly placed with respect to the transmission line shown in Figure 4a.

### 2.3. The Proposed DGS Unit Cell and Final Structure

**Figure 8:**
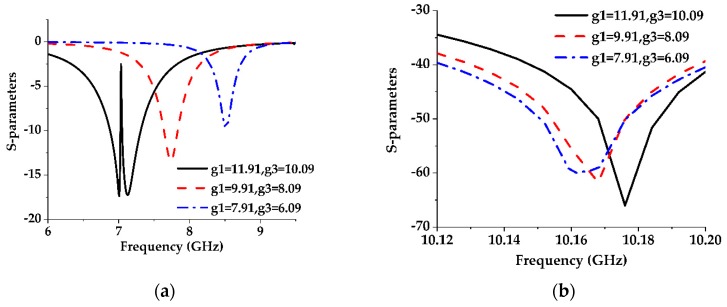
The effect of the lattice size on the S-parameters of the parallel 1-D EBG unit cell with a constant gap width of 1 mm. (**a**) S11; (**b**) S21.

The proposed DGS structure is of a 2-D multiperiod EBG geometry [27]. The analysis of the final structure begins with a 1-D pair of lattices etched in the metallic ground plane parallel to the feed line. This pair of EBGs can be thought of as a dumbbell-shaped DGS where the gap is removed. From Figure 8a, it is found that increasing the etched area of both the lattices results in a lower cutoff frequency which means that the series inductance increases due to the increase in the areas. Now unlike the conventional dumbbell-shaped DGS unit, the change in attenuation location is too small as shown in Figure 8b. This is because removing the gap from the dumbbell-shaped DGS causes it to separate into two rectangular EBG units. Each EBG unit can be thought of as a parallel LC resonator where the size of the etched area controls the inductance, and the distance between the edges controls the capacitance of the resonator. Increasing the etched area increases the series inductance, but the shunt capacitance decreases due to the increment of the distance between the edges. So, in this case, the resonance depends on the product of the two reciprocal terms (L and C) which slightly changes the location of the attenuation pole. In the present case, the product increases for the decrease in the etched area resulting in a lower passband frequency.

**Figure 9:**
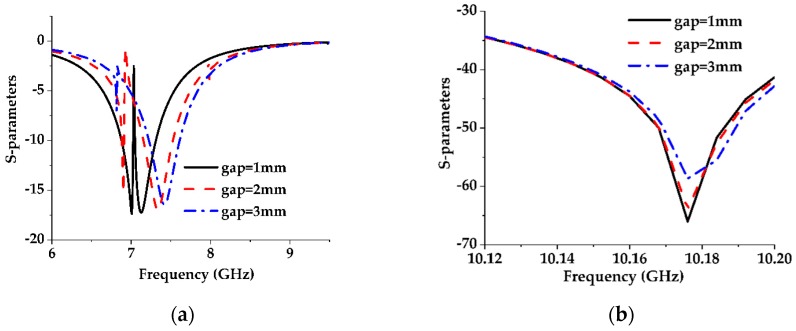
The effect of the gap width on the S-parameters with a constant lattice size of g1 = 11.91 mm, g3 = 10.09 mm. (**a**) S11; (**b**) S21.

**Figure 10:**
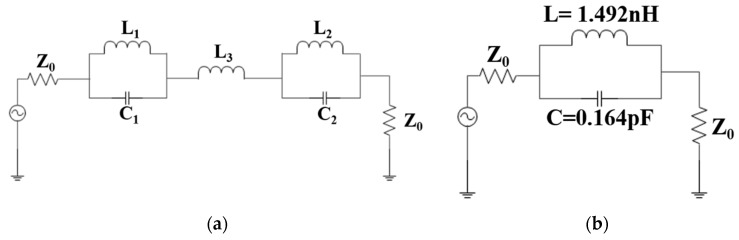
(**a**) The equivalent circuit of the proposed pair of EBG unit cells; (**b**) A simplified circuit.

On the other hand, if the etched area is kept constant and the gap width of the conventional dumbbell-shaped DGS is varied, the series inductance remains almost constant, and the shunt capacitance varies. For the proposed design, there is no such gap distance and hence, varying the distance between the two EBG units while the etched area is kept constant results in no change in the inductance value as well as no change in the shunt capacitance value. That’s why the location of the attenuation pole does not change as shown in Figure 9b. There is a slight change, however, in the cutoff frequency which is due to the inductance value introduced as a result of the narrow strip of copper between the two units (Figure 9a). So, for the EBG structure consisting of one pair of unit cells, the equivalent circuit can be imagined as two parallel LC resonators connected with an inductor as shown in Figure 10a. This circuit can be simplified to a single LC resonator as given in Figure 10b which is the equivalent to the circuit of the conventional dumbbell-shaped DGS structure.

#### Parameter Extraction

In order to apply the proposed pair of EBG unit cells, the equivalent circuit parameters need to be extracted. To extract the parameters of the EBG configuration, the values of some dimensions such as g1, g3, and g5 need to be fixed. The effects of these parameters on the frequency characteristics have been discussed earlier. The dimensions of these parameters have been chosen as the dimensions of the final fabricated prototype parameters which are, g1 = 11.91 mm, g3 = 10.09 mm, and g5 = 1 mm. The linewidth of the microstrip line has been chosen to be the characteristic impedance of 50 ohms for the simulation. To describe the cutoff and attenuation pole characteristic simultaneously, the circuit should give performances of low-pass and band-stop filter at the same time. So, the simulation result can be matched to the one-pole Butterworth-type low-pass response which has a 3 dB cutoff frequency at 6.424 GHz and an attenuation pole near 10.176 GHz. The series reactance value shown in Figure 5 can be easily calculated by using the prototype element value of the one-pole Butterworth response which is provided in various references [28,29]. The parallel capacitance value can be extracted from the attenuation pole location. Finally, the following equations, fulfilling the requirements of the Butterworth low-pass response, have been used to extract the series inductance and shunt capacitance value as shown in Figure 10b:

(1) $$C=\frac{\omega_{c}}{Z_{0}g_{1}}\cdot \frac{1}{{\omega_{0}}^{2}-{\omega_{c}}^{2}}$$

(2) $$L=\frac{1}{4\pi^{2}{f_{0}}^{2}C}$$

where,
$\omega_{c}$ is the angular cutoff frequency,
$\omega_{0}$ is the angular resonance frequency, and
$g_{1}$ is the prototype value of the Butterworth-type low-pass filter

Finally, a 2-D EBG which is formed by two 1-D proposed EBGs is designed and etched at the ground plane of the patch antenna with the stub-notch configuration to improve the performance of the antenna. The equivalent circuit of the final EBG structure is the same as the equivalent circuit of the 1-D EBG structure, and the simplified circuit is the same as shown in Figure 5. It is observed that the loading of the proposed 2-D multiperiod EBG with the stub-notch configuration results in increased bandwidth and is more matched.

### 2.4. Lumped Element Circuit

**Figure 11:**
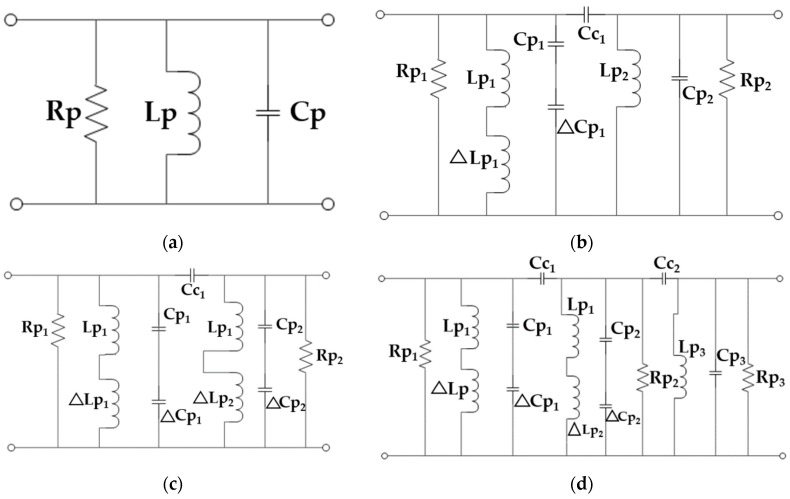
The equivalent circuit of various configurations of the antenna. (**a**) Simple patch antenna; (**b**) Patch antenna with inset; (**c**) Patch antenna with inset and single stub; (**d**) Patch antenna with inset and all stubs.

The equivalent lumped element circuit of a simple rectangular patch antenna can be designed as a parallel RC resonator which is based on the cavity model as shown in Figure 11a [22]. The loading of the insets means the insertion of two parallel slots into the patch increases the current path. The increment of the current path results in an additional series inductance (ΔLp1). An additional series capacitance (ΔCp1) has also been added to the circuit due to the slots. So, the final equivalent circuit is a combination of two RLC (A circuit combination of resistive(R), capacitive (C) and inductive (L) elements) resonators connected by a coupling capacitor Cc1 as shown in Figure 11b, and this design gives a dual-band frequency response [30]. The addition of a single stub, whose length is not equal to a quarter of the wavelength offers inductive and capacitive impedances to the higher and lower resonance frequencies, respectively. So, the loading of a single stub can result in an equivalent circuit with the addition of the capacitive impedance ΔCp2 and inductive impedance ΔLp2 to the circuit (Figure 11c). After the two stubs at each side of the center stub have been placed, another RLC resonator forms as shown in Figure 11d with a coupling capacitor (Cc2). Therefore, a triple resonance frequency has been observed. To ignore this resonance, the notches are inserted and the distance between the side stubs and the center stub has been increased. For this reason, the notches increase the current path which in turn increases the series inductance and the coupling capacitance. The value of the coupling capacitance can be ignored because of its low value with respect to the inductance of the third RLC circuit. Therefore, the simplified equivalent circuit will be the same circuit as that shown in Figure 11b with different parameter values.

**Figure 12:**
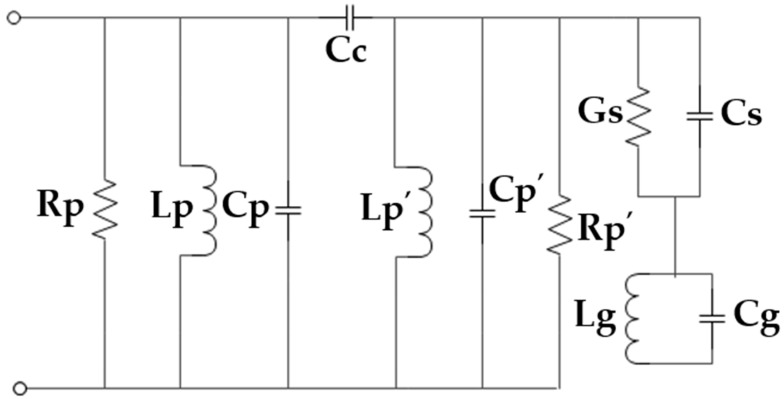
The complete lumped element circuit of the proposed antenna.

The complete lumped element circuit of the antenna can be found by connecting the coupling parameters of the dielectric substrate and the equivalent circuit of the DGS. As in the patch antennas, the dielectric material is used to separate two metallic layers of parallel combinations of capacitance, Cs and conductance Gs which can be assumed in the shunt between these two layers, as Gs is considered due to dielectric loss. Finally, the DGS section is connected in series with this parallel combination to form the complete lumped equivalent circuit of the antenna which is shown in Figure 12.

## 3. Results and Discussion

**Figure 13:**
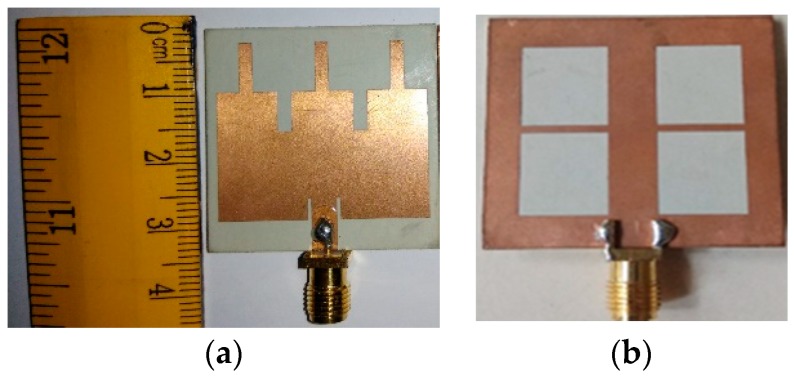
A fabricated prototype of the proposed antenna (**a**) The front side; (**b**) The back side.

**Figure 14:**
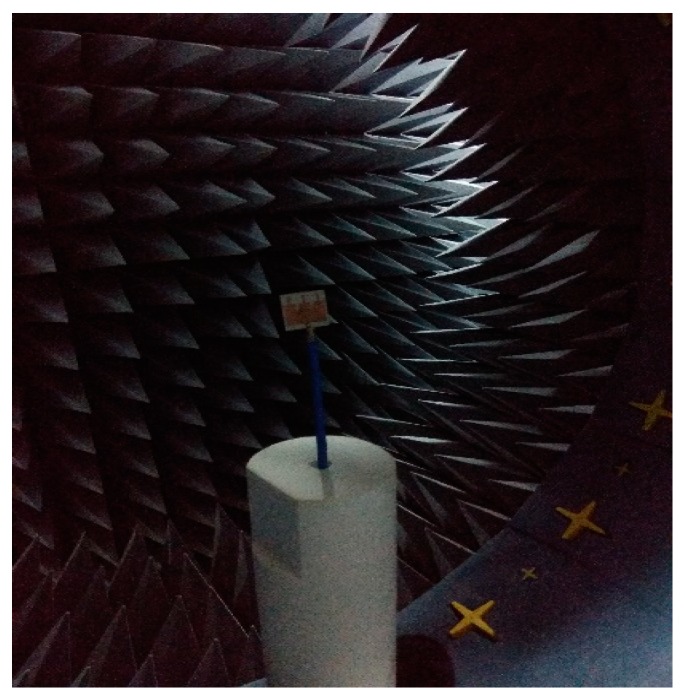
Satimo setup of the fabricated prototype.

**Figure 15:**
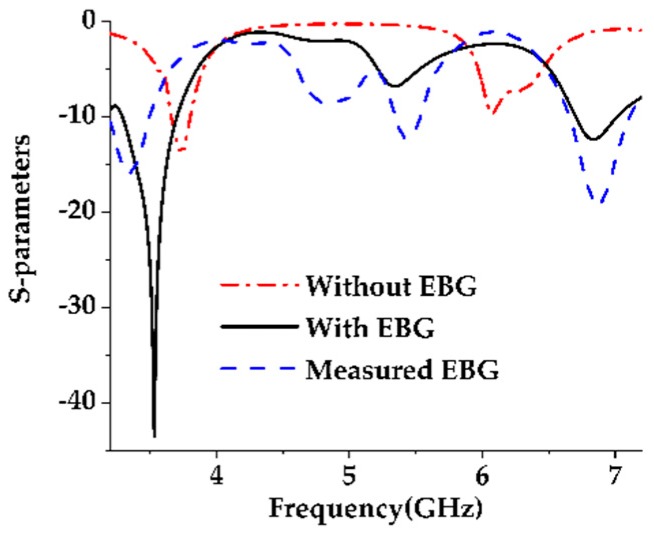
Comparison of the S-parameters of the EBG and Non-EBG configurations.

**Figure 16:**
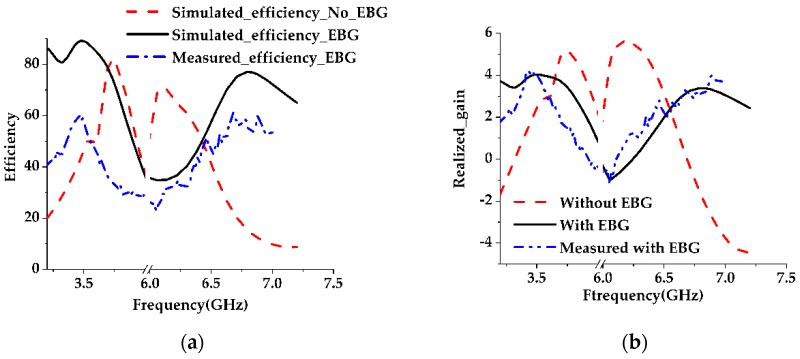
Comparison between EBG and non-EBG configurations. (**a**) Efficiency; (**b**) Realized gain.

The simulation and modeling of the proposed antenna are done using a commercially available CST Microwave studio. A prototype of the final EBG loaded design is fabricated and tested. The S-parameter is measured using a performance network analyzer (PNA) series vector network analyzer (E8362C 10 MHz–67 GHz) and the radiation characteristics, efficiency, and realized gain are measured using a Satimo Star-Lab near-field antenna measurement system. The photography of the fabricated prototype and its Satimo setup is shown in Figure 13 and Figure 14, respectively. The measurement begins with the placement of the antenna under test (AUT) in the middle of a circular arc on the test bed which consists of 16 separate receiving antennae as shown in Figure 14. This system measures the radiated power of the antenna in the near field region for computing the far field values of the AUT. The receiving antennae are spread out evenly across the arch. To get the 3D radiation pattern, the creation of a full 3D scan which has been done by rotating the AUT horizontally by 360 degrees is required [31]. The S-parameters of the final patch antenna with the EBG loaded and without the EBG is given in Figure 15. It has been found that because of the DGS structure, the bandwidth of the final proposed antenna increased. The efficiencies and realized gains of the desired passband frequencies are also increased which are shown in Figure 16a,b respectively. The measured data is in good agreement with the simulated result except for the result of the efficiency. This is because of the fabrication tolerance and also some small defects which were introduced during the soldering of the subminiature version A (SMA) connector which cannot be seen in Figure 13. Moreover, the loss of the radio frequency (RF) feeding cable is not considered during the simulation. A comparison between the different parameters of the present work and some previous work are given in Table 2. Though the antenna is bigger than other designs mentioned in the Table, it gives a wider bandwidth with better impedance matching that is one of the important requirements of 5G. Another important fact should be noted that implementing DGS in the ground plane shifts the passband frequency slightly to the lower side and results in a gain reduction. This may be due to the increased bandwidth because the relation between the gain and bandwidth are reciprocal to each other or it may be due to the resonating property of the DGS slots which is a common problem of this technique and has also been observed in previous research work [9].

**Figure 17:**
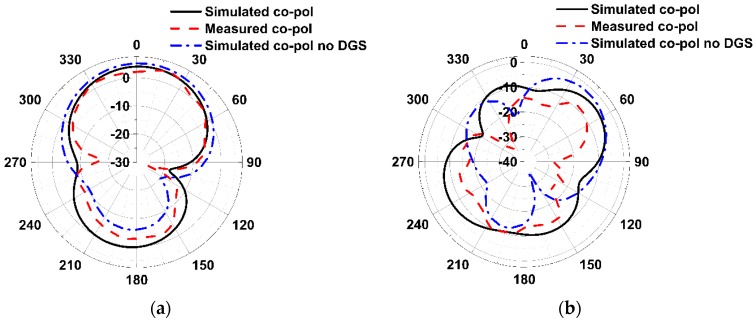
The radiation patterns in the YZ-plane. (**a**) 3.532 GHz, 3.736 GHz (no_DGS); (**b**) 6.835 GHz, 6.074 GHz (no_DGS).

**Figure 18:**
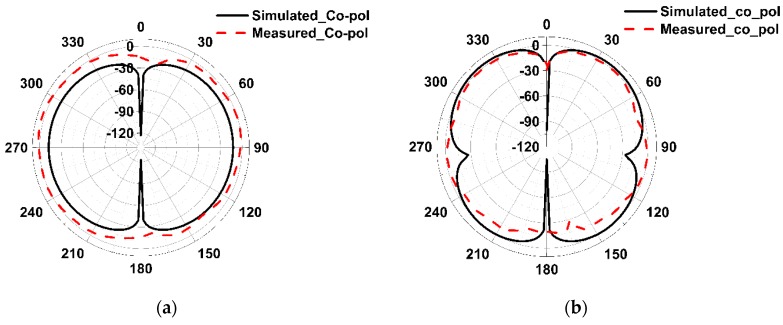
The radiation patterns in the XZ-plane. (**a**) 3.532 GHz; (**b**) 6.835 GHz.

**Figure 19:**
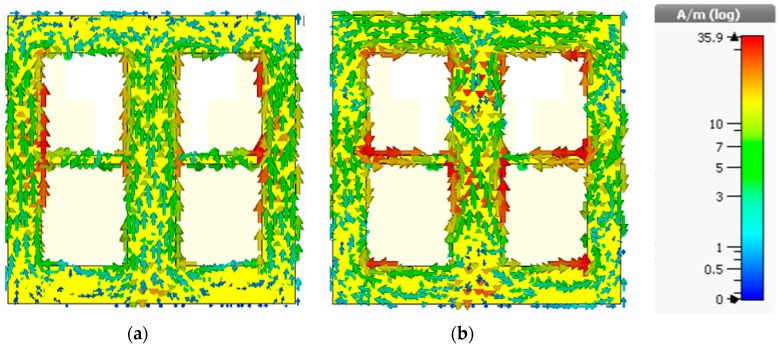
The current distribution of the ground plane of the antenna. (**a**) 3.532 GHz; (**b**) 6.835 GHz.

The radiation patterns in the YZ-plane and XZ-plane for the lower passband and upper passband frequencies with and without DGS (only YZ-plane) are shown in Figure 17 and Figure 18, respectively. From the radiation pattern in the YZ-plane, it can be observed that the EBG structure influences the patterns in the backward direction for both passband frequencies. This is because the slots in the ground plane are itself resonators and in the high-frequency region the effect is worse, which is clear from the current distribution plot of the ground shown in Figure 19.

That’s why a notch has been formed in the pattern of the higher passband frequency after implementing the EBG structure which was not present earlier. However, the radiation patterns are not in the same direction which is a common problem of the patch antenna. But the EBG configuration has little effect on it because after loading this structure, the increment of the main beam directions of the radiation patterns for the two passband frequencies is only 12 degrees.

## 4. Conclusions

The effect of DGS structure on the stub-loaded slot antenna is investigated here and has come to the conclusion that using DGS in conjunction with other performance-enhancing designs can lead to a better solution for the 5G technology. In the present work, it gives a wider bandwidth while maintaining a high gain and better impedance matching. These achievements are appropriate to fulfill the requirements of massive IoT services that will be one of the prime concerns of 5G. Though the miniaturization level is negligible which is shown in Table 2, it resonates in the desired 5G frequency spectrum where both licensed and unlicensed frequency bands are utilized. However, further investigation is needed in the field of DGS to reduce its back radiation and to experiment with other design methodologies so that it can play an important role in 5G technology.

## Figures and Tables

**Table 1:** Detailed Dimensions of the proposed antenna.

Parameters	Value (mm)	Parameters	Value (mm)
l_1_	33.5	w_5_	1.9
l_2_	19.17	w_6_	0.5
l_3_	7.38	w_7_	2.1
l_4_	6	g_1_	11.91
w_1_	33.5	g_2_	5.58
w_2_	9.1	g_3_	10.09
w_3_	12.91	g_4_	5.81
w_4_	3.68	g_5_	1

**Table 2:** The comparison between the results of some previous work with the present one (here λ represents the wavelength of the corresponding lower resonant frequency).

Antenna	Description	Antenna Dimension at the Lower Passband Frequency	Percentage Bandwidth	Gain (dB)	Efficiency (%)
[9]	Slit loaded antenna with DGS	(0.33 λ × 0.23 λ)	6.5 (2.45 GHz) 4.18 (3.5 GHz) 4.97 (5.28 GHz)	4.72 6.2 3.8	- - -
[13]	A dual-band highly miniaturized patch antenna with DGS	(0.16 λ × 0.15 λ)	- -	−1.7 (2.43 GHz) 2.4 (5.2 GHz)	30 81
[11]	Microstrip patch antenna for radiolocation using DGS with improved gain and bandwidth	(0.28 λ ×0.28 λ)	3.2 (5.9 GHz) 3.5 (9.1 GHz) 3.7 (10.4 GHz)	2.5 6.1 5.3	78.89 82.33 67.85
[32]	Dual-band slim microstrip patch antennas (no DGS)	(0.19 λ × 0.32 λ)	4 (1.9 GHz) 2.2 (3.5 GHz)	1 2.4	28 35
[33]	Slot loaded rectangular patch antenna on a glass-reinforced epoxy laminated inexpensive substrate	(0.09 λ × 0.12 λ)	81.72 (0.460 GHz) 6.99 (4.5 GHz)	6.6 (dBi) 9.7 (dBi) Average gain	85 88
[15]	Dual-band miniaturized microstrip slot antenna for wireless local area network (WLAN) applications	(0.16 λ × 0.32 λ)	1.673 (2.4 GHz) 2.2 (5.83 GHz)	2.76 1.7	52 31
[Present Work]	Dual-band slot antenna without DGS	(0.42 λ × 0.42 λ)	3.75 (3.736 GHz) 1.98 (−8 dB) (6.076 GHz)	5.164 5	82.15 70.61
	Dual-band slot antenna with DGS	(0.4 λ × 0.4 λ)	12.49(3.532 GHz) 4.49 (6.835 GHz)	4.02 3.38	88.2 76.88
